# ASO Author Reflections: The Impact of DCIS on Positive Margin Rates

**DOI:** 10.1245/s10434-024-15217-9

**Published:** 2024-03-28

**Authors:** Hemali Chauhan, Hutan Ashrafian, Zoltan Takats, Daniel Richard Leff

**Affiliations:** 1 Department of Surgery & Cancer, Imperial College London, London, UK; 2 Breast Unit, Charing Cross Hospital, Imperial College NHS Trust, London, UK

## Past

Anecdotally, surgeons regularly attribute a greater risk of margin positivity with ductal carcinoma in situ (DCIS) compared with its invasive counterpart. Although confirmed in the literature, previous reports grossly underestimate the risk of margin positivity with invasive breast cancer (IBC) containing a DCIS component (IBC + DCIS).^1^ We suspect that this is the result of the limitations of previous work, which focused on patient-level rather than margin-level analysis since studies evaluated diagnostic core biopsy data (DCIS present vs. DCIS absent) or patient-level data held in National databases (DCIS component in the primary tumour vs. no DCIS component in the primary tumour) rather than a detailed margin-level analysis (i.e., DCIS present at the margin vs no DCIS present at the margin) to characterise and validate the histopathological relationships. This study delineates the relative risk of IBC + DCIS compared with pure IBC (without a DCIS component) on margin positivity through detailed margin level interrogation.

## Present

Clinicopathological details were examined from 5454 margins from 909 women. Margin level interrogation included granular detail into the extent, pathological subtype, and grade of disease at each resection margin. Compared with pure IBC, the relative risk of a positive margin with IBC + DCIS is approximately ninefold, significantly higher than previous estimates and regardless of whether Association of Breast Surgery (UK) or Society of Surgical Oncology—American Society Radiation Oncology (USA) margin width criteria were applied. The relative risk was 8.76 (95% confidence interval [CI] = 6.64–11.56), applying Association of Breast Surgery guidelines, and 8.44 (95% CI = 6.57–10.84), applying the Society of Surgical Oncology—American Society for Radiation Oncology guidelines.^2^ This margin-level methodology is believed to represent the impact of DCIS more accurately on margin positivity in IBC.

## Future

Surgeons should pay particularly close attention to demographic and clinicopathological factors that are associated with DCIS margin positivity, such as young age, multifocal disease, microcalcifications, and comedonecrosis on the diagnostic core biopsy.^2^ These factors can be calculated into a unique, individualised risk score, which can be discussed with patients before surgery to allow informed clinical decision making. There is a plethora of intraoperative margin assessment tools being developed, and it is critical that these tools can accurately diagnose DCIS to optimise oncological margin control in vivo.

## References

[1] Langhans L, Jensen M-B, Talman M-LM, Vejborg I, Kroman N, Tvedskov TF (2017). Reoperation rates in ductal carcinoma in situ vs invasive breast cancer after wire-guided breast-conserving surgery. JAMA Surg..

[2] Chauhan H, Jiwa N, Nagarajan VR (2024). Clinicopathological predictors of positive resection margins in breast conserving surgery. Ann Surg Oncol..

